# Left ventricular assist device implantation and surgical repair in advanced congenitally corrected transposition of the great arteries

**DOI:** 10.1093/ehjcr/ytag004

**Published:** 2026-01-19

**Authors:** Wei Xie, Yi Ge, Hailong Cao

**Affiliations:** Department of Cardiac Surgery, Zhongda Hospital, School of Medicine, Southeast University, 87 Dingjiaqiao, Nanjing 210009, Jiangsu, China; Department of Cardiac Surgery, Zhongda Hospital, School of Medicine, Southeast University, 87 Dingjiaqiao, Nanjing 210009, Jiangsu, China; Department of Cardiac Surgery, Zhongda Hospital, School of Medicine, Southeast University, 87 Dingjiaqiao, Nanjing 210009, Jiangsu, China

**Keywords:** Congenitally corrected transposition of the great arteries (ccTGA), Left ventricular assist device (LVAD), Heart failure, Surgical repair

A 59-year-old male with a 10-year history of recurrent chest tightness and palpitations, markedly aggravated over the past month, now in INTERMACS profile 2 and NYHA class IV heart failure, with peripheral oedema and unable to perform a 6-minute walk test. His presentation was complicated by atrial fibrillation (*Panel A*), cardiogenic shock requiring continuous norepinephrine and epinephrine, and laboratory evidence of severe congestion (BNP 4915 pg/mL, hyperbilirubinaemia).

Pre-operative imaging confirmed congenitally corrected transposition of the great arteries (ccTGA) with severe functional right ventricular outflow tract (RVOT) obstruction, atrial and ventricular septal defects, severe mitral regurgitation (*Panel B–D,*  Supplementary material online, *Videos S1–3*). Echocardiographic measurements indicated that the free wall thickness was 10 mm for the functional left ventricle (morphological right ventricle) and 7.5 mm for the functional right ventricle (morphological left ventricle), with end-diastolic volumes of 194 and 66 mL, respectively. Right heart catheterization revealed high systemic morphologic right ventricular pressure [92/0 (32) mmHg] and the catheter could not be advanced across the severely stenotic RVOT. Notably, while heart transplantation remains the gold standard for end-stage heart failure, the patient was not considered a candidate for immediate transplantation due to severe pulmonary hypertension.

Given progressive decompensation and failed maximal medical therapy, the heart team determined that mechanical circulatory support was required. While options such as extracorporeal membrane oxygenation can provide short-term life support, they are often associated with a reduced quality-of-life and are difficult to maintain long-term. Considering that the functional left ventricle (morphological right ventricle) was severely dilated, a left ventricular assist device (LVAD) was selected to assist it by augmenting its ejection function, thereby maintaining cardiac function over a longer duration.

The heart team performed midline sternotomy under cardiopulmonary bypass, with implantation of the Corheart 6 LVAD into the functional left ventricle (morphologic right ventricle) (*Panel E* and *F*). Concomitant procedures included a modified Maze procedure with left atrial appendage closure for atrial fibrillation, RVOT muscle resection and pulmonary valve commissurotomy to relieve the severe RVOT obstruction, patch repair of the atrial septal defect and ventricular septal defect, and ring annuloplasty of the mitral valve. The tricuspid valve had good leaflet coaptation intra-operatively and did not require intervention. After completion of the procedures and careful adjustment of LVAD pump speed, the patient was successfully weaned off cardiopulmonary bypass.

Post-operatively, the patient was extubated at 16 h and managed with inotropic support and cautious diuresis to control the large systemic right ventricular volume and to optimize LVAD pre-load and after-load. Anti-coagulation was started with enoxaparin and then switched to warfarin, with a target international normalized ratio of 1.8–2.5. Rhythm management combined the surgical Maze procedure, short-term amiodarone and temporary pacing, and the patient achieved early rhythm stabilization (*Panel G*). He was discharged on post-operative day 15 with marked functional improvement (*Panel H-I*). At 2-month follow-up, he demonstrated a 6-minute walk distance of 650 m, and reported sustained quality-of-life gains.

This case confirms that LVAD implantation in ccTGA with advanced heart failure is technically achievable even in critically ill adults with complex anatomy, when combined with targeted structural repairs such as RVOT relief, closure of septal defects and valve annuloplasty.

**Figure:**
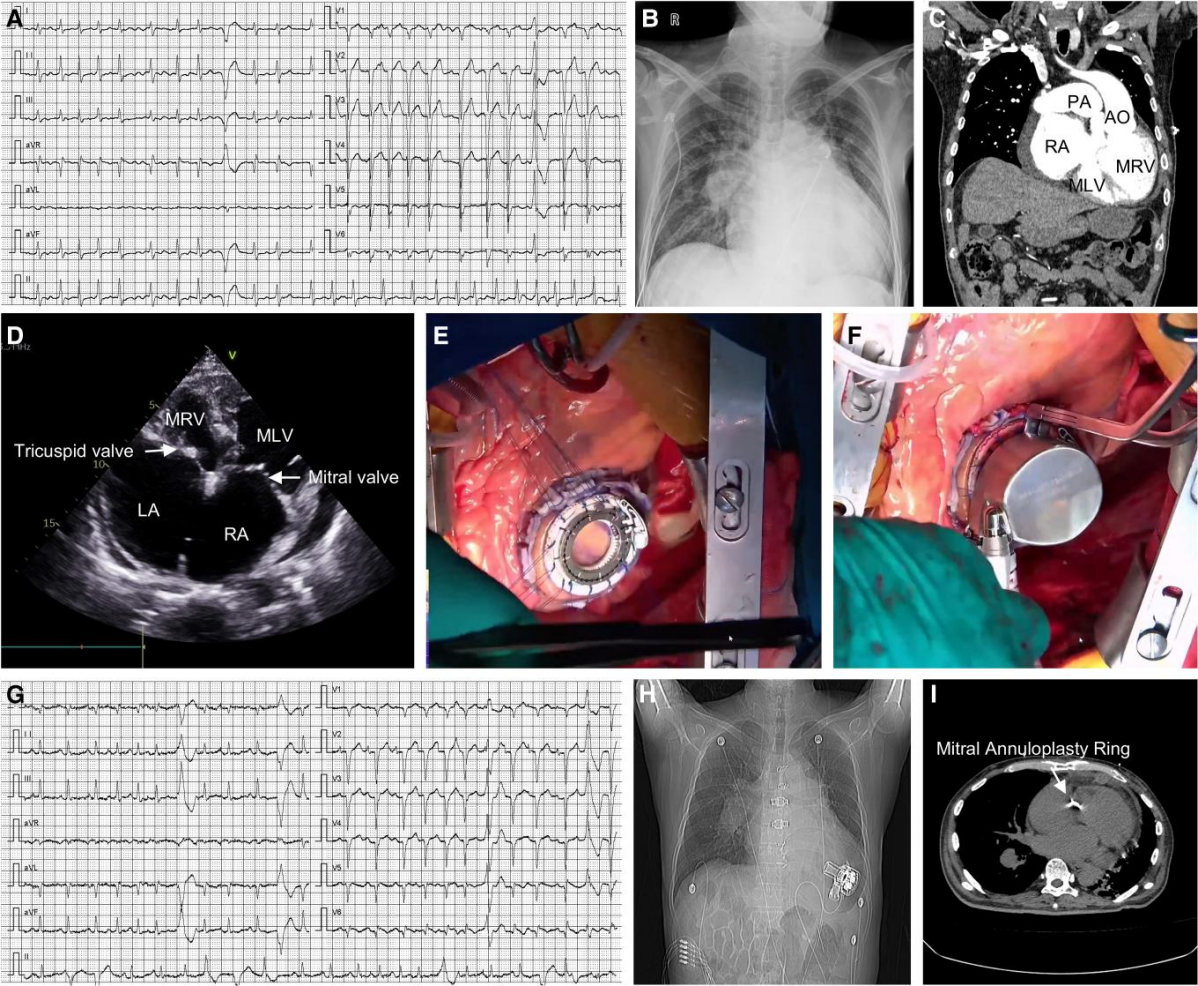


## Supplementary Material

ytag004_Supplementary_Data

## Data Availability

Data available on request.

